# Challenges and perspectives of selenium supplementation in Graves’ disease and orbitopathy

**DOI:** 10.1007/s42000-019-00133-5

**Published:** 2019-11-13

**Authors:** Tomasz Bednarczuk, Lutz Schomburg

**Affiliations:** 1 Department of Internal Medicine and Endocrinology, Medical University of Warsaw, ul. Banacha 1a, 02-097 Warsaw, Poland; 2 Institute for Experimental Endocrinology, Charité-Universitätsmedizin Berlin, Suedring 10, 13353 Berlin, Germany

**Keywords:** Graves’ disease, Graves’ orbitopathy, Selenium, Dietary supplements, Hyperthyroidism, Antithyroid drugs

## Abstract

**Purpose:**

This review focuses on the results of prospective randomized clinical trials and the conclusions from respective meta-analyses in order to summarize experiences with adjuvant selenium (Se) supplementation in Graves’ hyperthyroidism and orbitopathy, while identifying ambiguous findings and highlighting important open research issues.

**Methods:**

We searched the PubMed and the Cochrane Central Register of Controlled Trials (CENTRAL) through May 31, 2019, for relevant studies.

**Results:**

The available evidence concerning routine use of Se in the treatment of Graves’ hyperthyroidism remains ambiguous. Results of meta-analyses, including ten RCTs, suggest that adjuvant Se supplementation can enhance the restoration of biochemical euthyroidism. Unfortunately, these results must be judged cautiously owing to several important limitations. Moreover, an in-depth analysis of relevant long-term clinical measures of therapeutic success (such as remission rate after antithyroid drug treatment), besides surrogate markers (for example, hormone or autoantibody concentrations), is generally missing. Based on a single study, Se supplementation is recommended in patients with mild orbitopathy of short duration because it may decrease inflammation and eye-specific symptoms while also achieving a marked improvement in disease-specific quality of life. The effects of Se supplementation on moderate-to-severe orbitopathy remain as yet unknown.

**Conclusions:**

Additional randomized clinical trials with clinically relevant endpoints are urgently needed to further aid in clinical decision-making, including better stratification of Graves’ disease patients, who are most likely to benefit from Se supplementation.

## Introduction

Graves’ disease (GD) is an autoimmune disease caused by autoantibodies (aAb) binding to and activating the thyroid-stimulating hormone receptor (TSHR). Chronic stimulation of the TSHR results in a hyperactive thyroid gland, largely independent of the concurrent thyroid hormone status of the patient. GD is the most common cause of hyperthyroidism in iodine-sufficient countries. Unfortunately, there has been little progress in the treatment of Graves’ hyperthyroidism (GHT) over the last few decades and optimal therapy remains a subject of debate. There are three major treatment options for thyrotoxicosis: (i) antithyroid drugs (ATD), e.g., methimazole, carbimazole, or propylthiouracil; (ii) radioactive iodine (RAI); and (iii) subtotal or total thyroidectomy [1]. All three methods are effective, but all three also have significant and therapy-specific side effects. In Europe, ATD remains the principal treatment regimen, although it is associated with high rates of relapse (40–60%), depending mainly on the patient’s baseline autoantibody levels and age [2]. The severity of GHT is variable, while about 25–50% of GD patients will develop some degree of ophthalmopathy [3]. Treatment of Graves’ ophthalmopathy (GO) remains a challenge, and both GHT and GO significantly affect patients’ quality of life (QoL), persisting for many months after therapy [4]. Hence, there is a clear unmet medical need to search for improved treatment modalities for GD.

Like other autoimmune conditions, GD is a complex disorder, with a combination of genetic and environmental risk factors involved in its pathogenesis [5]. These include modifiable nutritional risk factors [6]. Epidemiological studies suggest that low dietary selenium (Se) intake is associated with increased susceptibility and severity of GD [7]. Selenium is an essential trace element, which is incorporated into selenoproteins and which has a wide range of pleiotropic effects, ranging from antioxidant and anti-inflammatory effects to the production and degradation of thyroid hormones [8–10]. It has thus been hypothesized that Se supplementation may have a beneficial role in the treatment of GHT and GO by ameliorating the autoimmune inflammation [11, 12]. Despite the availability of results from several clinical intervention trials, the effectiveness of Se supplementation as adjuvant therapy and the parameters modulating the potential success of supplemental Se in GD remain largely unknown and poorly characterized. One major limitation of our current understanding is the lack of large intervention studies, as the available insights are mainly deduced from trials enrolling fewer than 100 patients, necessitating the attempt to conduct meta-analyses on results from not always fully comparable trials. This short review focuses on the results of several prospective randomized clinical trials (RCTs) and the conclusions from relevant meta-analyses with the aim of summarizing recent experiences with Se supplementation in clinical practice while identifying ambiguous findings and highlighting important open research issues.

## The effects of Se supplementation on medical treatment in Graves’ hyperthyroidism

Adjuvant Se supplementation during ATD treatment could have an effect on (i) restoration of euthyroidism, (ii) remission rate after treatment, and (iii) QoL.

### Restoration of euthyroidism

Several trials have investigated the effect of add-on Se supplementation on the control of hyperthyroidism in GD patients treated with ATD (methimazole) with conflicting results (Table 1). The first prospective, randomized, double-blinded, placebo-controlled study with Se in GD was conducted by Calissendorff et al. in Sweden, an area with known Se deficiency [13]. In this study, newly diagnosed GHT patients were randomized to receive ATD using a block-and-replace regimen, in combination with either Se (200 μg/day as yeast tablets) or placebo for 9 months. The authors observed improved biochemical control of thyroid dysfunction. The main biochemical findings were a reduction of FT4 at 18 and 36 weeks and an increase of TSH at 18 weeks [13].

**Table 1:** Selected randomized clinical trials on the use of Se and methimazole in Graves’ hyperthyroidism

Characteristics	Study			
	Calissendorff J et al.	Wang L et al.	Leo M et al.	Kahaly GJ et al.
Country	Sweden	China	Italy	Germany
Case/control	19/19	21/20	15/15	35/35
Selenium status of GD patients	Se deficient	Not accessed	Se replete	Se replete
Intervention	Selenious yeast tablet 200 μg/day	Sodium selenite 200 μg/day	l-Selenomethionine 166 μg/day	Sodium selenite 300 μg/day
Methimazole treatment	Block and replace regimen	Titration regimen	Titration regimen	Titration regimen
Placebo control	Yes	No	No	Yes
Duration of therapy (months)	9	6 + 12 (follow-up)	3	6 + 3 (follow-up)
Main findings	Se supplementation can enhance biochemical restoration of hyperthyroidism	Se supplementation can enhance the effect of antithyroid drugs: restoration of euthyroidism and remission rates.	No adjuvant role of Se in the short-term control of hyperthyroidism was observed.	Supplemental Se did not affect response or recurrence rates in GD. Se-related side effects were not observed.

Similar results were reported by Wang and collaborators, who performed a prospective study in China. Patients with recurrent GHT were allocated to receive ATD (methimazole) alone or in combination with sodium selenite (200 μg/day) for 6 months [14]. The method of allocating participants was quasi-random, dividing patients by date of consultation. After 2 months of treatment, levels of FT4 and FT3 were decreased in the Se group compared with those in the control group. Moreover, a significantly lower level of TSHR antibodies (TRAb) was detected in patients receiving Se supplementation [14].

On the other hand, two recent intervention studies reported conflicting findings. Kahaly et al. conducted a double-blind, placebo-controlled, randomized supplementation trial in Se-sufficient GHT patients in Germany [15]. In addition to ATD (methimazole in a titration regimen), patients received either sodium selenite (300 μg/day) or placebo for 6 months. During treatment, serum concentrations of thyroid-related hormones and thyroid autoantibodies decreased, albeit without statistically significant differences between the two groups or placebo- and selenite-treated patients [15].

These results are in line with the negative findings in a Se-sufficient Italian cohort, in which newly diagnosed GHT patients were randomly assigned to treatment with ATD (methimazole in a titration regimen) alone or in combination with Se (166 μg/day as selenomethionine) [16]. During the first 3 months of therapy, Se supplementation had no effect on clinical findings or thyroid hormone levels.

Recently, Zheng et al. performed a meta-analysis to evaluate the effects of Se supplementation on thyroid function in GHT patients treated with ATD by combining the results from the different studies that are currently available [17]. The researchers identified ten suitable RCTs involving a total of 796 GD patients. Most of the studies (7 out of 10) were conducted in China, and the remaining studies were performed in Europe (Sweden, Italy, and Germany, as described above). The intervention groups received various Se compounds: selenized yeast tablets (seven studies), sodium selenite (two studies), or L-selenomethionine tablets (one study), in dosages ranging from 100 to 300 μg/day. Only two RCTs were conducted as double-blinded and placebo-controlled trials. Only the three European trials measured serum Se levels and/or biomarkers sequentially to determine the baseline level and/or supplementation effects on total serum Se or selenoprotein expression. The duration of the trials was relatively short: patients received MMI ± Se for a maximum of 9 months, and the duration of follow-up ranged up to a maximum of 12 months.

The results of the meta-analysis suggest a beneficial effect of Se in terms of biochemical control of thyroid hyperfunction. Patients receiving ATD and supplemental Se attained euthyroidism faster than the control group. There was a statistically significant decrease in FT3 and FT4 levels in the Se-treated groups at 3 months and 6 months, but not at 9 months. Se supplementation was consistently associated with significant increases in TSH levels at 6 months, but not at 3 and 9 months of treatment. Moreover, the meta-analysis suggests that Se supplementation may effectively reduce TRAb levels at 6 months, but not at 9 months [17]. These findings largely agree with the results from systematic reviews and meta-analyses reporting that Se supplementation is associated with a significant decrease in the levels of thyroid peroxidase antibodies (TPOAb) in the treatment of chronic autoimmune thyroiditis [18]. However, the lack of consistency over time and the variable effect strengths indicate that several important parameters modulating the adjuvant therapy response of GHT to supplemental Se are not yet understood.

The molecular targets of Se in patients with GHT are unknown, but some direct dampening effects on the activity of plasma cells would explain the overlap of beneficial effects on circulating Ab concentrations observed in both GD and chronic autoimmune thyroiditis. Alternatively, supplemental Se may modify the autoantigens of thyroid cells by affecting their processing, quality control in the endoplasmic reticulum and targeting the membrane, the MHC-dependent presentation on the cell surface, or other redox-sensitive posttranslational modifications. However, none of these mechanisms has convincingly been shown to be Se-responsive in respective cell culture work or animal models, implying that the immune system–thyroid interaction is simultaneously affected at several critical steps, involving both the endocrine gland and the effector cells of the immune system [11, 12]. Moreover, to what degree improved expression of the Se-dependent deiodinase isozymes contributes to the therapeutic effects in GHT by improving thyroid hormone activation in target cells and controlling their degradation, respectively, remains to be studied in sufficiently large intervention trials with patients exhibiting different Se baseline status.

The findings presented in the meta-analysis [17] must therefore be interpreted with care due to the several important limitations of the underlying studies: (i) substantial heterogeneity between trials was detected in some important aspects of the groups of patients and the treatment regimen; (ii) all of the RCTs included were of small size only, applying different Se compounds, dosages, and treatment periods; (iii) most of the studies did not report on Se status either before, during, or at the end of the interventions. Finally, an in-depth analysis of relevant long-term clinical measures of therapeutic success, besides surrogate markers like autoantibody levels or hormone concentrations, is generally lacking. At present, we are thus left with some promising results, albeit without an understanding of the mechanisms involved or how best to identify those patients most likely to benefit from supplemental Se.

### Remission/relapse rates after treatment

The results of prospective intervention studies evaluating the remission/relapse rates after ATD treatment with adjuvant Se supplementation are conflicting. Wang and collaborators demonstrated higher remission rates in patients with recurrent GHT supplemented with Se (52.3 vs. 25.0%), thus concluding that Se supplementation can enhance the effect of antithyroid therapy [14]. In contrast, Kahaly et al. reported almost equal remission rates in the Se and the placebo groups (41 vs. 45%, respectively) [15]. The authors confirmed that serum levels of FT4, FT3, and TRAb, prevalence of GO, thyroid volume, and MMI starting dose were associated with the response to ATD therapy. Elevated serum concentrations of Se and selenoprotein P convincingly verified the successful intervention and allowed for a time-resolved analysis of supplementation effects on the individual Se status; however, the cases of increased serum Se status were not related to the patients’ response or recurrence rates. On the other hand, the study groups were again relatively small in both trials and the time periods of active intervention were rather short, thus warranting larger and longer intervention studies to better determine a potential prevention of relapse after successful treatment. Again, data from larger studies may help to stratify for those patients who will most likely respond positively to adjuvant Se supplementation after achieving euthyroidism.

### QoL of patients during treatment

The supplementation study conducted by Calissendorff et al. assessed QoL with meaningful questionnaires assessing depression, anxiety, and self-rated disease symptoms before medication and Se supplementation were started and subsequently after 9 months of treatment [13]. In general, the depression and anxiety scores were similar in the groups receiving placebo or Se. Notably, however, the authors reported a significant correlation between recovery from the disease and the clinical symptoms in the Se group only, which was not the case in the placebo group. Again, the groups may not have been of sufficient size and the trial may have been too short to better pinpoint health-relevant effects of the supplemental Se. Some comparable intervention trials with similar dosages of Se have reported on improved QoL specifically in the Se-treated groups, e.g., in the treatment of mild GO [19], or as combination with coenzyme Q10 in community-dwelling elderly people [20], whereas other small studies failed to detect any positive effects of Se on QoL. Further studies with a larger and more heterogeneous group of thyroid patients monitoring potential effects of supplemental Se on QoL in GD are clearly indicated to identify whether baseline Se status, disease activity or severity, ATD therapy, or other parameters are important modulators of the Se effects and who may most likely benefit from a supplement.

## The effects of Se supplementation on GO

The role of Se in GO was studied in only one prospective, randomized, placebo-controlled trial, which recruited patients with mild active eye disease of short duration (<  7 months) [19]. One hundred fifty-nine GO patients were randomized to receive Se supplementation (200 μg/day of sodium selenite), pentoxifylline, or placebo for 6 months and were evaluated over a total time period of 12 months [19]. The overall responses were significantly better in the Se group compared with those in placebo, including a decrease in inflammation and eye-specific symptoms along with a marked improvement in disease-specific QoL (mentioned above). Furthermore, very few patients treated with Se progressed to more severe disease. Unfortunately, the Se status of the study population was not determined and the results have not as yet been confirmed in an independent study. Moreover, the effects of Se supplementation on moderate-to-severe GO and on the emergent condition of sight-threatening GO remain unknown. This lack of verification studies is most unfortunate, as other treatment options in GO are few and the disease burden of affected patients is very high.

## Guidelines

The American Thyroid Association (ATA) and the European Thyroid Association (ETA) found insufficient evidence to suggest Se supplementation in the management of Graves’ hyperthyroidism [21, 22]. Based on a single study [19], the ETA/European Group on Graves’ Orbitopathy (EUGOGO) are recommending a 6-month period of Se supplementation to be implemented in patients with mild GO of short duration because it may improve eye manifestations and QoL and prevent GO progression to more severe forms (strong recommendation, moderate quality of evidence) [23]. This recommendation is not shared by the ATA, as US American patients are usually not Se deficient and thus not in need of supplementation.

## Areas of uncertainty

Despite the published guidelines, Se supplementation is widely used in daily clinical practice and its supplemental intake is not restricted to mild GO. A questionnaire-type study performed in 2016 among Italian endocrinologists demonstrated that 20% of respondents would add Se to ATD therapy in a hypothetical 42-year-old female with GHT. When the case was changed to include active GO, up to 25% of the respondents would recommend Se supplementation [24]. More recently, members of the ETA were invited to participate in an online survey investigating the use of Se in GD either with or without GO [25]. This study showed that 38% of the responders recommend Se supplementation in GHT and almost all responders (94%) recommend Se supplementation in GO (both in patients with mild and in those with moderate to severe GO).

Importantly, most European clinicians do not take the individual or regional Se status into account when deciding on whether to start Se supplementation or not. This widespread use is criticized in view of the fact that the evidence appears limited for a beneficial therapeutic effect of Se in thyroid disease, apart from mild EO [26]. On the other hand, there are no reports of any harm or side effects of supplemental Se when taken by European subjects at the recommended dosages (50–200 μg/day). In our estimation, the health risks associated with a pronounced Se deficit outweigh the worries, while correcting a Se deficit likely provides additional health benefits. Clinical studies have indicated elevated risks for certain health issues in subjects with low Se status beyond thyroid disease only, e.g., higher odds of death from severe disease [27] or polytrauma [28], increased incidence of gastrointestinal or hepatocellular cancer [29, 30], or even cardiovascular events [31].

Although strong evidence is as yet lacking that Se supplementation results in clinical improvement in GD or GHT (other than mild GO), it appears nevertheless reasonable to ensure adequate Se intake and avoid severe Se deficiency. However, the following aspects should be taken into consideration in order to minimize potential risks and provide maximal benefits.

### Evaluation of Se status prior to supplementation

Daily Se intake depends on individuals’ dietary pattern, and in Europe, grain, local fruits, and vegetables are poor sources of the essential trace element, this placing vegetarians in particular need of supplemental Se [32–35]. European subjects consuming an omnivore diet can easily improve their Se status by a more balanced choice of nutrition, i.e., by increasing their egg, milk, meat, or sea fish consumption, while vegetarians can turn to South American nuts, imported grain from, e.g., the USA, vegetarian seafood items, and/or supplements. Meanwhile, in several European countries, Se-enriched fodder is normally used in animal farming, while sea fish and other sea fruits, many of which are Se rich, are often consumed, and South American nuts, which are thought to potentially contain large amounts of Se [33], are available. Of interest, the only European country taking this systemic deficiency seriously enough for active intervention is Finland, where all fertilizers have been Se-enriched since 1985 [32]; side effects from this nationwide supplementation have not been reported until now.

Besides nutrition and residing in a known geographical area with Se deficiency, there are other potential causes of an individual showing reduced Se metabolism and low Se status, some of these being chronic disease, inflammation, liver disease, and pregnancy. Irrespective of the underlying cause of Se deficiency, the quantitative analysis of a bio-sample is recommended, since such an analysis avoids insecurity and provides a solid estimation on personal Se status. This type of diagnostic analysis can be conducted applying several matrices, e.g., serum, plasma, blood, nail, or hair. For the purposes of optimal handling, reproducibility, and availability of reference ranges, blood, serum, or plasma samples are the preferred bio-samples, with total Se usually being determined. Alternatively, selenoproteins can be measured, i.e., glutathione peroxidase 3 activity from serum or plasma, glutathione peroxidase 1 activity from blood cells, or selenoprotein P from serum or plasma [36]. The latter biomarker may constitute the most meaningful measure of Se status [37] and can be assessed by calibrated ELISA tests and controlled with commercially available reference material [38]. Such standardization is unfortunately not available for any of the selenoenzyme activity tests, restricting their use mainly to comparative analyses of larger cohorts of samples.

Total Se can be determined from a blood, serum, or plasma sample by a variety of techniques, including atomic absorption spectroscopy, inductively coupled plasma-atomic emission spectroscopy, inductively coupled plasma-mass spectrometry, reflection X-ray fluorescence, and gas chromatography. These methods are reliable and robust if the setup is optimized, but require sophisticated and expensive equipment that is not easily and everywhere available, highly qualified personnel, and in-depth long-term experience. Quantification of total Se by a service provider or of selenoprotein P by a commercial ELISA test-kit may currently be the easiest and least complicated ways to determine the Se status and identify subjects exhibiting a Se deficit.

### Differences in the regimen of Se supplementations

In published supplementation trials, both inorganic (usually sodium selenite) and organic (usually selenomethionine and sometimes Se-enriched yeast) Se compounds were used as intervention agents. At present, there are few intervention trials comparing different supplements in the same setting in order to determine whether supplementation effects on the expression of selenoproteins differ and whether one Se compound offers a better therapeutic effect or is safer than another. Some authors point out that Se levels in subjects receiving organic Se may show a steady rise during treatment, whereas those supplemented with inorganic Se supplements reach a plateau after several months, suggesting a safer approach during chronic supplementation [39]. On the other hand, selenite appears to be distributed into the organs and body fluids more efficiently and universally than selenomethionine [40]. Theoretically, selenomethionine can be directly inserted into all proteins in response to AUG codons and does not only serve as a Se source for the biosynthesis of selenoproteins; thus, unlimited uptake may ensue, eventually reaching toxic concentrations [8, 41]. Selenite, on the other hand, can be directly used for selenoprotein biosynthesis or be excreted, upon surplus intake after hepatic conversion, via the urine [42]. However, in practice, there are no reports of toxicity from selenomethionine, Se yeast, or selenite intakes in subjects with poor Se status when the recommended dosages of 50–200 μg/day are not surpassed.

Nevertheless, besides the nature of the selenocompound, the quality of the supplements can be of utmost importance, as epidemic cases of selenosis have been reported from supplements that were wrongly formulated and exceeded the intended dosages by several orders of magnitude [43].

The ideal dosage of Se supplementation also remains unclear, especially in cases of short-term adjuvant treatment attempts vs. long-term chronic nutritional supplementation. The recommended dietary allowance (RDA) for adults varies between 55 and 70 μg/day, depending on the average Se status of different populations [44]. In order to reach an optimal serum Se concentration of ~ 100 μg/L, or to safely avoid a severe Se deficit, supplemental Se in dosages of 50–200 μg/day should be adequate. The daily dosage of the supplement should not exceed 300 μg/day, as, otherwise, in combination with dietary intake, the upper tolerable level of Se intake of 400 μg/day in the USA or 300 μg/day in the EU is easily surpassed. Finally, the requirements to reach optimal Se status may differ from subject to subject due to polymorphisms in selenoprotein genes and a number of individual parameters, e.g., health status, age, sex, or current medication.

**Fig. 1:**
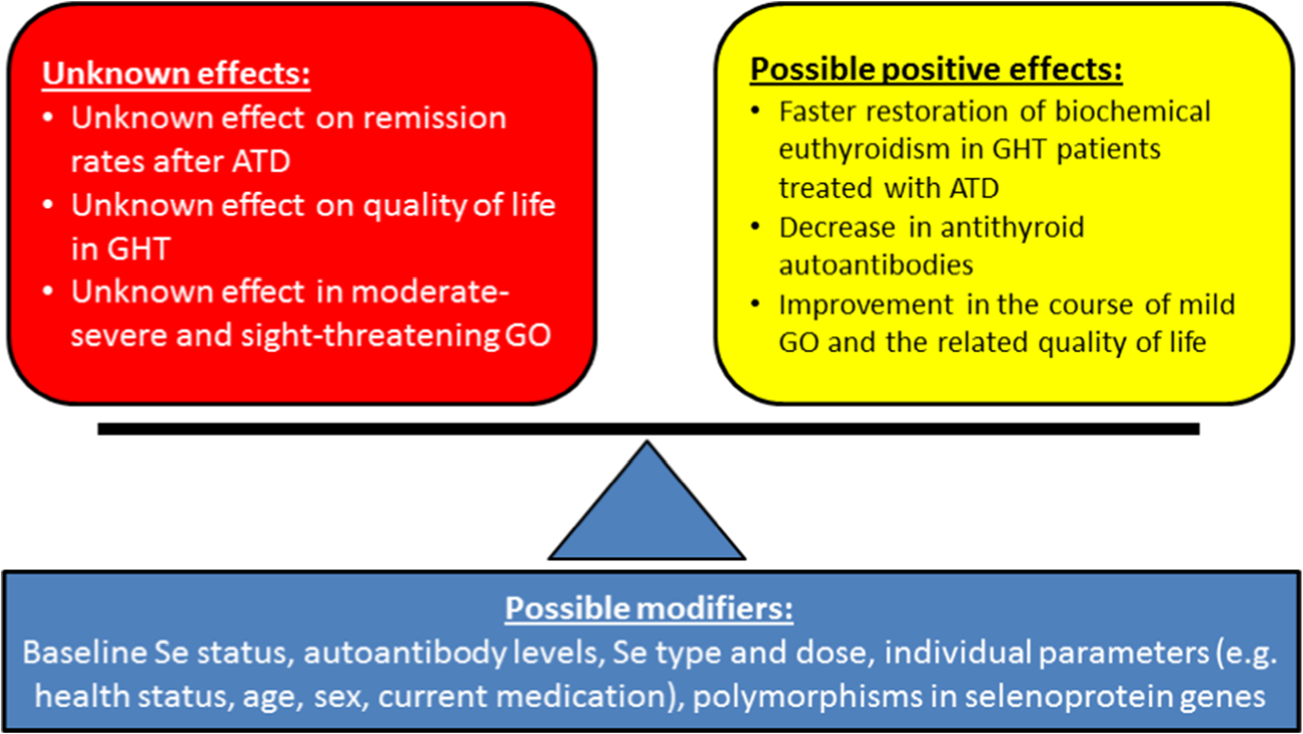
Selenium supplementation in Graves’ disease. The trace element selenium (Se) may be of high relevance for the treatment of patients with Graves’ disease (GD) and Graves’ orbitopathy (GO). At present, however, it cannot be predicted whether a given patient will benefit from supplemental Se in terms of preventing remission, improving quality of life, or reducing eye symptoms in moderate-or-severe GO (red box, left). On the other hand, supplemental Se may accelerate restoration of euthyroidism in combination with antithyroid drugs (ATD), decrease autoantibody titers, and improve mild GO symptoms and quality of life, along with a number of other health benefits (yellow box, left). Several modifiers of the Se effects are known, and a decision on potential supplementation with Se needs to balance these unknown and suspected effects, optimally in combination with monitoring the Se status of the particular patient

Future research better defining the relevance of these parameters to Se metabolism and highlighting how genetic, anthropometric, and environmental factors interact will broaden our understanding of both preventive and curative medicine (Fig. 1). One very instructive, as well as surprising, example came to light with the recent analysis of the role of lifelong Se intake in thyroid disease incidence rates in Shaanxi province, China, where two rural areas with > 3000 participants each were compared. The highly different soil Se concentrations were found to translate into an approx. twofold difference in Se status of the rural populations. This moderate difference in serum Se concentrations (mean, IQR; 103.6 [79.7, 135.9] vs. 57.4 [39.4, 82.1]), which very closely corresponds to the range seen in EU-wide studies, translated into an approx. twofold difference in thyroid disease incidence [45]. The in-depth analysis of the data highlighted the fact that the incidence of hyperthyroidism showed the typical sex-specific difference, being higher in females than in males. However, while the female incidences of hyperthyroidism were not affected by Se intake and were similar in the low Se area and in the area with a higher supply, the incidence of hyperthyroidism in males was markedly higher in the low Se area compared with that in the area with higher intake [46]. This finding indicates again that there are sex-specific differences in Se metabolism and its relation to human health [47]. Potential differences in Se metabolism in males and females need to be considered, while intervention studies should be conducted with both male and female participants. When taking gender into consideration, the current recommendations for optimal Se intake should probably also include weight as an important factor for determination of the “healthiest” dosage of Se. A weight- and purpose-adapted recommendation may be suitable as a starting point for future interventions, e.g., 1 μg/(kg × day) for chronic supplementation in preventive medicine for avoidance of Se deficits, and 2 μg/(kg × day) for disease-related interventions under medical guidance and monitoring, respectively. Such weight- and purpose-adapted Se intakes could also positively affect body weight development and measures of obesity, as Se deficiencies are more prevalent in overweight subjects and may contribute to a feedforward cycle in the context of “hidden hunger” for a certain micronutrient [48]. However, these notions and recommendations are mainly derived from theoretical considerations based on available study results and not deriving from respective clinical interventions. They should thus be considered as helpful suggestions for the design of future trials and their interpretation, but not yet as solid knowledge.

### Use of additional antioxidants

One of the first studies on patients with GD evaluated the effect of supplementation of a mixture of antioxidants, including vitamins C and E, beta-carotene, and Se (60 μg/day) in a group of patients treated with ATD [49]. The study was conducted in Croatia, a country with habitual low Se intake. Patients who received the additional supplementation with antioxidants attained a more rapid biochemical and clinical remission than those who received methimazole alone. These results were partially confirmed by a small study using an antioxidant combination of vitamins C and E, beta-carotene, Cu, Zn, Mn, and Se [50]. Thus, the role of additional vitamins and antioxidants apart from Se in ameliorating disease symptoms and supporting remission remains to be established in larger studies, preferentially in combination with close monitoring of the status of these parameters along with Se at time points before, during, and at the end of the intervention.

## Ongoing studies

Additional RCTs with clinically relevant endpoints are clearly needed to further aid in clinical decision-making. We are currently awaiting the results of the GRASS trial (GRAves’ disease Selenium Supplementation trial), which enrolled 492 patients with GHT in Denmark [51]. The aim of this study is to analyze the effects of Se supplementation (200 μg/day as Se-enriched yeast) in addition to standard treatment with ATD over a longer period of time (24 to 30 months). Endpoints of the study are response to ATD (restoration of euthyroidism, remission rate after treatment) and assessment of quality of life. Other registered trials (https://clinicaltrials.gov, https://www.cochranelibrary.com) include the following: “Selenium in Mild Thyroid Eye Disease in North America” (NCT02112643), “Oral Supplementation With Selenium in Patients With Mild Thyroid Orbitopathy” (NCT03891043), “The Effect of a New Antioxidant Combination (ASTED) on Mild Thyroid Eye Disease (TED)” (NCT02393183), “The Effect of a New Antioxidant Combination (ASTED) on Moderate to Severe Thyroid Eye Disease” (NCT02422368), “The Comparison of anti-inflammatory vs. supplement dose of selenium in mild Graves’ orbitopathy” (TCTR20180228005), and “Prospective Clinical Case-control Study of Selenium treatment for mild to moderate Graves’ ophthalmopathy” (ChiCTR-IOR-15005855). It is expected that especially the larger sizes of these trials in combination with the fact that some are being conducted outside Europe in areas of higher baseline Se status will allow for a better understanding of the mode of action of Se in GD (correcting a deficit vs. acting as a medication) and of the patients benefiting from Se supplementation. The results are therefore eagerly awaited by basic researchers, clinicians, and patients alike.

## Summary

- The available evidence concerning routine use of Se supplementation in the treatment of GD patients remains ambiguous [26]. Results of meta-analyses, including ten RCTs, suggest that adjuvant Se supplementation can enhance the restoration of biochemical euthyroidism. Unfortunately, these results must be judged cautiously owing to several important limitations, especially the lack of criteria for selecting the patients most likely to respond favorably. Although therapeutic use is recommended in the current ETA/EUGOGO guidelines, beneficial effects of Se supplementation in mild GO have been demonstrated in one RCT only and still need to be replicated.
- Despite published guidelines, Se supplementation is highly prevalent in the treatment of all thyroid diseases in daily clinical practice, both initiated by the treating physician or endocrinologist and patient-driven as self-medication. Negative side effects are not known in marginally supplemented European subjects when Se is taken in the recommended dosage range of 50–200 μg/day. An open discussion with the patient on the potential benefits and risks of supplemental intake, along with advice on the preferable dosage taking the patient’s Se status and body weight into consideration, appears to be the most responsible procedure, especially in view of the patients otherwise turning to uncontrolled self-medication.
- Additional RCTs will hopefully allow for a better stratification of GD patients who are most likely to benefit from Se supplementation as well as identifying the most suitable Se form and dosage for a safe and efficient adjuvant treatment.
